# Capillary Refill Time After Induction of General Anesthesia: A Pilot Study

**DOI:** 10.1213/ANE.0000000000007257

**Published:** 2024-10-10

**Authors:** Zbigniew Putowski, Szymon Czajka, Anna Szczepańska, Wojciech Szczeklik, Eduardo Kattan, Glenn Hernández

**Affiliations:** From the * Centre for Intensive Care and Perioperative Medicine, Jagiellonian University Medical College, Cracow, Poland; † Department of Anaesthesiology and Intensive Care, Faculty of Medical Sciences in Katowice, Medical University of Silesia, Katowice, Poland; ‡ Departamento de Medicina Intensiva, Facultad de Medicina, Pontificia Universidad Católica de Chile, Santiago, Chile.

A growing number of studies have emphasized the importance of peripheral perfusion assessment via capillary refill time (CRT) measurement in septic and other forms of circulatory shock.^1^ According to evidence derived from critically ill populations, CRT is a dynamic marker of peripheral perfusion and its normalization is correlated with tissue reperfusion. Similarly to distributive shock, general anesthesia (GA) is thought to be associated with systemic arterio- and venodilation, by reducing sympathetic activity caused primarily by propofol and anesthetic gases.^2^ Induction of anesthesia, during which large amounts of sedative and analgesic drugs are administered, should therefore affect vascular tone in a rapid fashion. As the local increase in tissue perfusion is often mediated by vasodilation, we hypothesized that the induction of anesthesia would be associated with shortening of the CRT. To test this hypothesis, we designed this pilot study in which a cohort of low-risk surgical patients were subjected to repeated CRT measurements immediately before and after GA induction.

## METHODS

This was an observational cohort study performed in the University Clinical Center in Katowice, Poland, between December 1, 2023 and May 15, 2024. The study was approved by the Bioethics Committee of the Silesian Medical University in Katowice (PCN/CBN/0052/KB/1/40/III/21/23) and written informed consent was obtained. Only adult, American Society of Anesthesiologists (ASA) ≤2, undergoing elective, noncardiac GA patients were eligible for the inclusion. Any severe cardiovascular disease, known peripheral artery disease, diabetes mellitus, and the requirement for vasoactive treatment during the induction of GA were treated as the exclusion criteria.

Standard dosing regimen for the induction of GA included: 1.5 µg/kg of fentanyl, 2 mg/kg of propofol, and 0.6 mg/kg of rocuronium. Intubation was performed approximately 3 minutes after propofol bolus. After intubation, the minimal alveolar concentration (MAC) of sevoflurane was targeted for 1.0.

CRT measurement was recorded at the palmar surface of the right or left index using a standardized method which has already been described.^3^ CRT measurements were recorded by a phone camera (IPhone 14 Pro) with a flashlight. Pressure on the finger was applied via a microscopic glass for 10 seconds, enabling the standardization of pressure. CRT value was verified by using a chronometer and through a video analysis by using the freeware Kinovea (www.kinovea.org).^4^

CRT measurements were made in 5 timepoints: (1) before the induction of GA (before the fentanyl bolus), (2) 1 minute after infusion of propofol, (3) 2 minutes after infusion of propofol, (4) 5 minutes after infusion of propofol, and (5) 10 minutes after infusion of propofol. Oscillometric method was used to measure mean arterial pressure (MAP), systolic blood pressure (SBP), and diastolic blood pressure (DBP), at each timepoints. Furthermore, heart rate (HR) was measured.

As this was a pilot study, no sample size calculation was performed. Instead, it was assumed that enrolling 20 patients would be sufficient for identifying any macroscopic trends in CRT. In this sense, the study should be regarded as exploratory.

**Figure.**
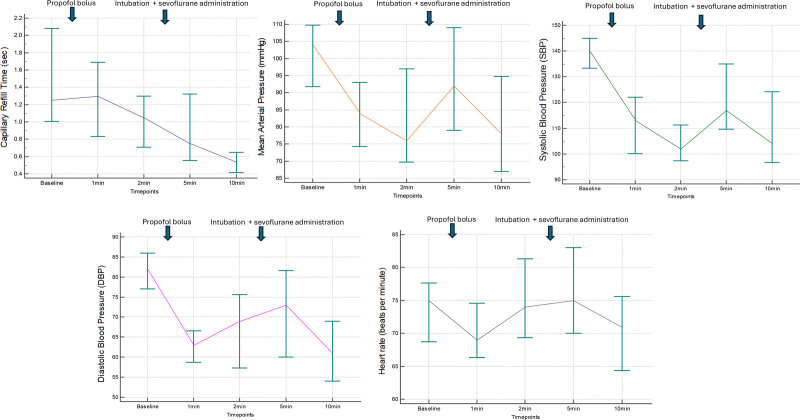
Consecutive CRT, MAP, SBP, DBP, and HR recordings during the induction of anesthesia. Values are presented as median with their interquartile ranges. Due to nonnormal distribution of baseline CRT, MAP, and HR, the ANOVA Friedman test was applied. ANOVA tests revealed that all variables changed significantly over time (*P* < .0001 for CRT, *P* = .00013 for MAP, *P* < .00001 for SBP, *P* = .0003 for DBP, and *P* = .02 for HR). Multiple comparisons tests showed that CRT, MAP, SBP, and DBP had a significantly lower value at 10 min compared to the baseline values (*P* < .05). Only HR did not exhibit the same relationship (*P* = .3). ANOVA indicates analysis of variance; CRT, capillary refill time; DBP, diastolic blood pressure; HR, heart rate; MAP, mean arterial pressure; SBP, systolic blood pressure.

Depending on normality or nonnormality of variable distributions, repeated measures analysis of variance (ANOVA) or ANOVA Friedman tests were used to detect significance in the consecutive CRT, MAP, SBP, DBP, and HR values. Post hoc multiple comparisons tests were also performed, but no correction for multiplicity was applied. Statistical calculations were performed by using MedCalc Software (v18.2.1.).

## RESULTS

No patients initially eligible for the study had to be excluded later due to the need for vasoactive treatment. In sum, there were 22 patients enrolled for the analysis. The median age was 49 years, with 17 (77.3%) female patients. Most of the patients (15/22) had no comorbidities and only 7 had a known chronic arterial hypertension. Of these, 1 patient continued beta-blocker and 2 patients continued Ca^2+^ channel blocker during the day of surgery (the remaining 4 subjects had their antihypertensive drugs withdrawn). As for the type of surgery, 11/22 (50.0%) underwent gynecological, 7/22 (31.8%) abdominal, 2/22 (9.1%) laryngeal and 2/22 (9.1%) spinal surgery. Changes in CRT, MAP, SBP, DBP, and HR are presented in the Figure. The median (interquartile range, IQR) CRT was 1.26 (1.0–2.1) at baseline and then changed to 1.30 (0.8–1.7), 1.05 (0.7–1.3), 0.75 (0.6–1.3), 0.54 (0.4–1.4) at 1, 2, 5, and 10 minutes respectively. The ANOVA Friedman test showed significant changes between consecutive measurements (*P* < .0001). In the multiple comparisons test, CRT at 10 minutes postinduction showed the highest difference from all prior measurements (−57.1% difference from the baseline CRT). At the same time, significant changes in blood pressure and heart rate were observed.

## DISCUSSION

In this pilot study, we showed that the induction of GA significantly reduced CRT, reaching the shortest duration at 10 minutes postinduction. Other studies with similar drug-dosing regimens have observed that GA induction is associated with a sudden decrease in systemic vascular resistance (SVR), consistent with our results in which we demonstrated peripheral vasodilation.^5^ Furthermore, anesthesia has been linked with modifying the skin vasoconstriction threshold, mediating perioperative hypothermia.^6^

Perfusion through skin vasculature, which is highly saturated with alpha-1 receptors, is grossly dependent on sympathetic stimulation.^7^ Excluding extreme thermal conditions, skin perfusion is generally well-maintained, and can be usually observed as CRT <2 seconds. However, when circulatory shock occurs and both neurological and vascular reactivity are intact, sympathetic activation causes vasoconstriction within various organs, including the skin. This is to redirect oxygen-carrying blood to more critical organs and sustain their function.^8^ Thus, one of the determinants of skin hypoperfusion is the degree of vasoconstriction. Indeed, in patients with anaphylactic or neurogenic shock, pathological vasodilation is often associated with augmented skin perfusion. Our findings are analogous to the above phenomenon: anesthesia-mediated vasodilation-enhanced skin perfusion.

While the direct impact on patient outcomes is uncertain in the perioperative care, there is evidence suggesting that a higher peripheral perfusion index (the pulsatile portion of flow derived from pulse oximetry: higher value indicates peripheral vasodilation and greater arterial inflow) is associated with fewer complications in surgical patients, perhaps due to correlation of skin perfusion with internal organs.^9^ Perioperative assessment of skin perfusion could therefore emerge as an interesting parameter to assess the adequacy of hemodynamic therapy. Macrohemodynamic optimization, through the administration of fluids, vasopressors, or inotropes, could be tailored in the light of tissue perfusion assessment, allowing to individualize therapy, and avoid detrimental effects of the fixed targets.^10^

As for the limitations of this study, the study had a small sample size (however, the results were consistent across the study subjects). Furthermore, cardiac output and SVR were not measured.

In conclusion, CRT is a rapidly changing hemodynamic parameter that is significantly influenced by the induction of GA, which tends to significantly reduce CRT.

Future studies should assess the relevance of these results and their relationships with concurrent hemodynamic and tissue perfusion changes during the perioperative setting.

## DISCLOSURES

**Conflicts of Interest:** None. **Funding:** None. **This manuscript was handled by:** Charles Emala, MS, MD.
